# Jumping for Dear Life with a Giant Neuron and a Single Receptor

**DOI:** 10.1371/journal.pbio.0040093

**Published:** 2006-02-28

**Authors:** Françoise Chanut

Flies are jumpy, prone to buzzing away at the slightest moving shadow or breeze. This reflexive “jump response” is a lifesaver for the tiny insects, whose only defense against predators is a quick escape. In flies, as in many animals, evolution has forged efficient reflex circuits that transmit nerve impulses from sensory organs to muscles within milliseconds.

The centerpiece of the fly's jump response circuit is the giant fiber. The large cell body of this prominent nerve cell resides in the brain and its axon extends into the fly's thorax. There, it connects with motor neurons that innervate the jump muscles in the leg and the flight muscles that power the wings. In the brain, the giant fiber receives visual inputs, caused by moving shadows, from the eyes while mechanical inputs, caused by wind or vibrations, come from the antennae.

Neurons communicate via specialized contact points called synapses, where they exchange electrical or chemical signals. The small molecule acetylcholine is a widespread chemical neurotransmitter in the fly brain that could mediate various steps in the jump response. Acetylcholine binds to a large variety of transmembrane receptors. In a new study, Amir Fayyazuddin and Hugo Bellen find that a membrane protein belonging to the nicotinic class of acetylcholine receptors, Dα7, is required within the giant fiber to link the jump response to sensory inputs.

Acetylcholine receptors clump together at neuron synapses, forming pores that open in the presence of extracellular acetylcholine. When a synapse fills with acetylcholine, the pores of the receiving neuron open and let in positively charged ions. A massive electrical impulse ensues that elicits signal release at the next synapse downstream. Fayyazuddin and his colleagues used a small piece of mobile DNA that had inserted next to the *Dα7* gene in the fly genome to generate several fly strains devoid of part or most of their *Dα7* gene. In the laboratory, scientists elicit the jump response in captive flies by simply turning off the light briefly. Normal flies jump while mutant flies stay put. The *Dα7* mutants failed to jump in response to the light-off signal, even though additional tests showed they had normal vision and locomotion.

In immobilized and partially dissected flies, scientists can fire up the giant fiber with an electrical stimulus and follow the nerve impulse all the way from giant fiber to twitching jump and flight muscles. Fayyazuddin and his colleagues found that in *Dα7* mutants, the flight muscles failed to respond to stimulation by the giant fiber. This result was not unexpected, as earlier observations had shown that a particular neuron that connects the giant fiber to flight muscles uses acetylcholine as its messenger. By contrast, in this assay, the jump muscle behaved normally in *Dα7* mutants. This was surprising, because jump precedes flight in the escape response, and the *Dα7* mutants have a clear jump deficit.

The researchers reasoned that the *Dα7* mutations silenced synapses farther upstream in the circuit. The Dα7 protein is abundant at brain synapses between the giant fiber and neurons that relay sensory inputs from the eyes and antennae. Acetylcholine is also found at these synapses, though its function has been unclear. Introducing an active *Dα7* gene in the giant fiber of mutant flies was sufficient to restore activation of the giant fiber. This result demonstrates that the giant fiber uses its Dα7 receptor (and acetylcholine) to trigger the jump reflex in response to sensory inputs.

Nicotinic acetylcholine receptors are notoriously difficult to study, because various family members can substitute for each other and attenuate the consequences of the loss of a single representative. It is therefore remarkable that disrupting the *Dα7* gene of *Drosophila* could have such a profound effect, especially on a behavior as vital as escape. The fly's Dα7 receptor may have evolved special properties that enhance the effectiveness of the escape reflex, properties that now warrant further investigations.

## 

**Figure:**
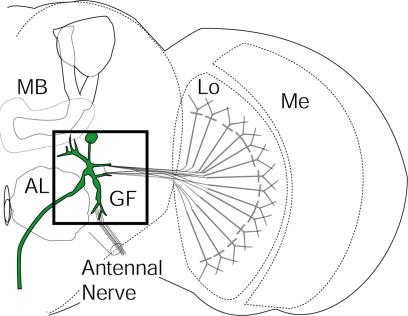
This schematic of the *Drosophila* brain shows the location of the giant fiber in relation to other brain regions (AL, antennal lobe; GF, giant fiber; Lo, lobula; MB, mushroom body; Me, medulla).

